# Micro-CT_vlab_: A web based virtual gallery of biological specimens using X-ray microtomography (micro-CT)

**DOI:** 10.3897/BDJ.4.e8740

**Published:** 2016-11-01

**Authors:** Kleoniki Keklikoglou, Sarah Faulwetter, Eva Chatzinikolaou, Nikitas Michalakis, Irene Filiopoulou, Nikos Minadakis, Emmanouela Panteri, George Perantinos, Alexandros Gougousis, Christos Arvanitidis

**Affiliations:** ‡ Hellenic Centre for Marine Research (HCMR), Gouves, Heraklion, Crete, Greece; § Institute of Computer Science (ICS), Foundation for Research and Technology - Hellas (FORTH), Science and Technology Park of Crete, Vassilika Vouton, Heraklion, Greece

**Keywords:** micro-CT, virtual lab, 3D visualisation, virtual galleries, cyberspecimens, digitised collections

## Abstract

**Background:**

During recent years, X-ray microtomography (micro-CT) has seen an increasing use in biological research areas, such as functional morphology, taxonomy, evolutionary biology and developmental research. Micro-CT is a technology which uses X-rays to create sub-micron resolution images of external and internal features of specimens. These images can then be rendered in a three-dimensional space and used for qualitative and quantitative 3D analyses. However, the online exploration and dissemination of micro-CT datasets are rarely made available to the public due to their large size and a lack of dedicated online platforms for the interactive manipulation of 3D data. Here, the development of a virtual micro-CT laboratory (Micro-CT_vlab_) is described, which can be used by everyone who is interested in digitisation methods and biological collections and aims at making the micro-CT data exploration of natural history specimens freely available over the internet.

**New information:**

The Micro-CT_vlab_ offers to the user virtual image galleries of various taxa which can be displayed and downloaded through a web application. With a few clicks, accurate, detailed and three-dimensional models of species can be studied and virtually dissected without destroying the actual specimen. The data and functions of the Micro-CT_vlab_ can be accessed either on a normal computer or through a dedicated version for mobile devices.

## Introduction

X-ray microtomography (micro-CT) is a non-destructive X-ray imaging technology which creates high-resolution three-dimensional data. In the past years, micro-CT has been increasingly used in several biological research fields, such as taxonomy (e.g. Faulwetter et al. 2013, Stoev et al. 2013, Fernández et al. 2014, Akkari et al. 2015), evolutionary and developmental research (e.g. Marxen et al. 2007, Metscher 2009, O’Connor et al. 2010) and functional morphology (e.g. Nickel et al. 2006, Curtis et al. 2009, Wojcieszek et al. 2012, Schulz-Mirbach et al. 2013, Paterson et al. 2014). This imaging technology gives the user the option to visualise both external (morphology) and internal (anatomy) characteristics of a specimen, to rotate and virtually dissect the resulting three-dimensional (3D) representation, as well as to perform 3D measurements and analyses. In addition, it seems that staining with heavy metals (e.g. iodine, PTA), which is often used in micro-CT studies to increase tissue contrast, do not cause any irreversible changes to the morphology of the specimen (but not much is yet known about the effects on the tissue or cellular level, Faulwetter et al. 2013). Therefore, micro-CT imaging is especially suitable for creating accurate, virtual representations of valuable and irreplaceable natural history museum specimens.

In an increasingly computerised world, the digitisation and dissemination of biodiversity information is growing rapidly, and specialised repositories for a variety of data types exist (e.g. NCBI databases for molecular data, OBIS and GBIF for biogeographical data, Biodiversity Heritage Library for digitised literature, Morpho-D-Base for media files, or MorphoSource for 3D data). However, although many micro-CT datasets of biological specimens are being created and published (e.g. Lenihan et al. 2014, Akkari et al. 2015, Landschoff et al. 2015, Tessler et al. 2016), a standardised practice for metadata annotation or publication of such datasets is absent (Faulwetter et al. 2013, Stoev et al. 2013). The reasons for this are: a) micro-CT datasets typically are large (several GB per dataset), thus storage space and manpower for adequate curation need to be reserved; b) existing community standards for biodiversity data (e.g. DarwinCore) are not fully suitable for the description of micro-CT datasets, and users have to rely on ad-hoc solutions. In addition, an online repository for micro-CT datasets would require not only a standardised annotation of datasets to make them searchable and retrievable, but ideally, online tools would allow the user to get a preview of the contents of the database or to directly interact with the dataset without having to download it or to install a suitable rendering software — a challenge for developers and maintainers of such a system, given the large dataset size.

The Micro-CT virtual laboratory (Micro-CT_vlab_) was created to contribute to the abovementioned needs. The Micro-CT_vlab_ offers the user a collection of virtual 3D specimens which are annotated with metadata and can be interactively displayed and retrieved through a web-based application. It allows the user to search for adequate datasets and to interact with the 3D models by using a series of online tools. These developments were supported by two complementary research projects: a) the EU FP7 project SYNTHESYS3, in the framework of which protocols, workflows and data management practices as well as online dissemination methods for virtual specimens were researched and developed, and b) the ESFRI LifeWatchGreece infrastructure, which supported the integration of the data into a large semantic infrastructure and provided the technical infrastructure and developments for the Micro-CT_vlab._

## Project description

### Design description

*Data creation, processing and publication within the Micro-CT _vlab_*

**Figure 1.**
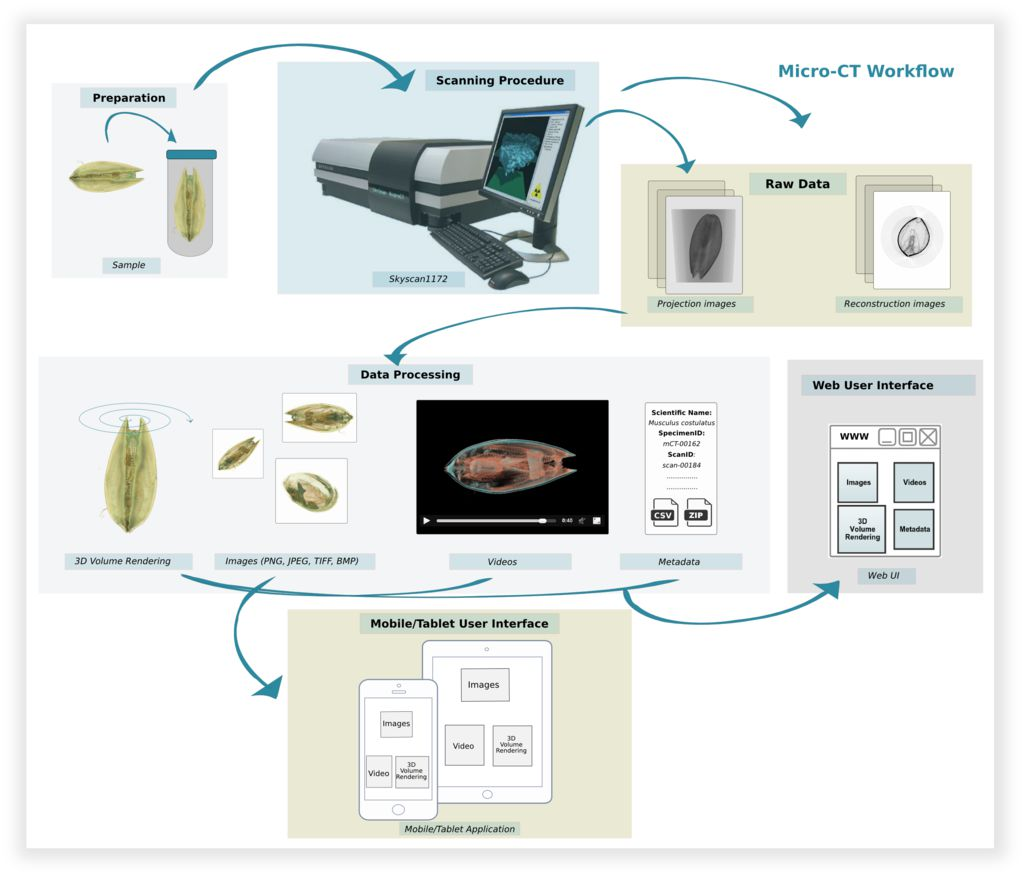
Data creation, processing and publication within the Micro-CT_vlab_.

The implementation of the Micro-CT_vlab_ takes into account all steps and procedures which contribute to the creation of the final dataset, from the preparation of the specimen to scanning parameters and post-processing of the data (Fig. 1). To be able to adequately represent this workflow in a virtual enviroment, a detailed analysis of the every-day workflow in the laboratory was created, identifying all actions and “objects” (persons, specimens, files) associated with each step. This workflow was then translated into a protocol which was henceforth followed for each dataset creation (Faulwetter et al. 2015). The preparation of the selected specimen is the first step for the creation of a micro-CT dataset (e.g. fixation of the specimen, contrast enhancement technique, re-/de-hydration of the specimen, etc.). Then, the specimen is scanned with appropriate scanning parameters to create projection images (greyscale images where grey values represent different X-ray densities) which are finally reconstructed via a dedicated software into cross-section images. Currently, all scans for the Micro-CT_vlab_ are performed using a Skyscan 1172 microtomograph at the Hellenic Centre for Marine Research (HCMR), but scans from other institutions or instruments can likewise be stored in the system. The abovementioned reconstructed cross-section images can be used to create a three-dimensional volume rendering of the specimen, to create screenshots and videos of the specimen rendered in 3D or to perform quantitative analyses. All steps and procedures are documented in detail using a set of specific metadata terms. For each dataset published through the Micro-CT_vlab_, preview images and videos of the dataset are created using volume rendering softwares such as CTVox or Drishti (Limaye 2012). These, along with a short description, help the user to understand the dataset's content. The metadata are stored in the central metadata catalogue of LifeWatchGreece (Data Services) and are displayed dynamically for each dataset published in the Micro-CT_vlab_.

*Micro-CT_vlab_ Feature Description*

**Figure 2.**
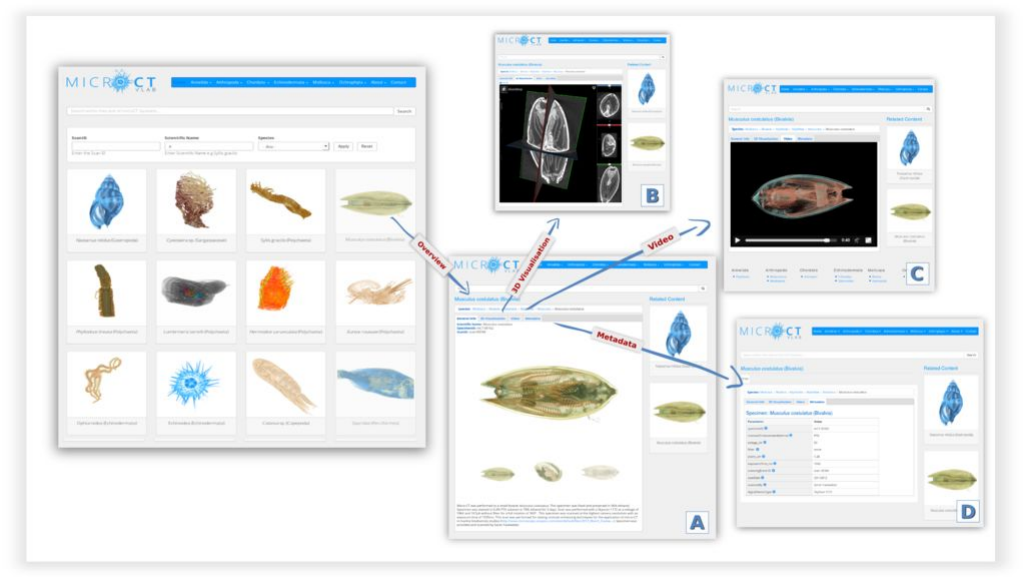
**Micro-CT_vlab_ web interface.** Each micro-CT dataset includes four tabs which allow the user: A) to get an overview of the dataset, B) to interact with the 3D representation, C) to watch a video of the model and D) to view the related metadata.

The Micro-CT_vlab_ is a web application compatible with all major web browsers, and available also as a mobile version. So far, 17 micro-CT datasets have been published, representing a selection of marine species scanned with different parameters. On the main page, scans are presented as a preview of images accompanied by the title of the dataset (Fig. 2A). A search function enables the user search for micro-CT datasets by e.g. species name, taxonomic classification or terms occurring in the dataset description. When any of these micro-CT datasets is selected by the user, the dataset details are displayed in four tabs (described in detail below), featuring: a) an overview page; b) an interactive tool for manipulating the 3D representation; c) a preview video and d) metadata for the dataset. Currently, the system does not feature a native option for the download of raw data (cross-sections), but several of the micro-CT datasets are available for download via the Dryad repository.

The **General Info** tab contains a description of the dataset, the taxonomic classification of the specimen and a series of 3D images which serve as a preview to the dataset (A).

**Figure 3.**
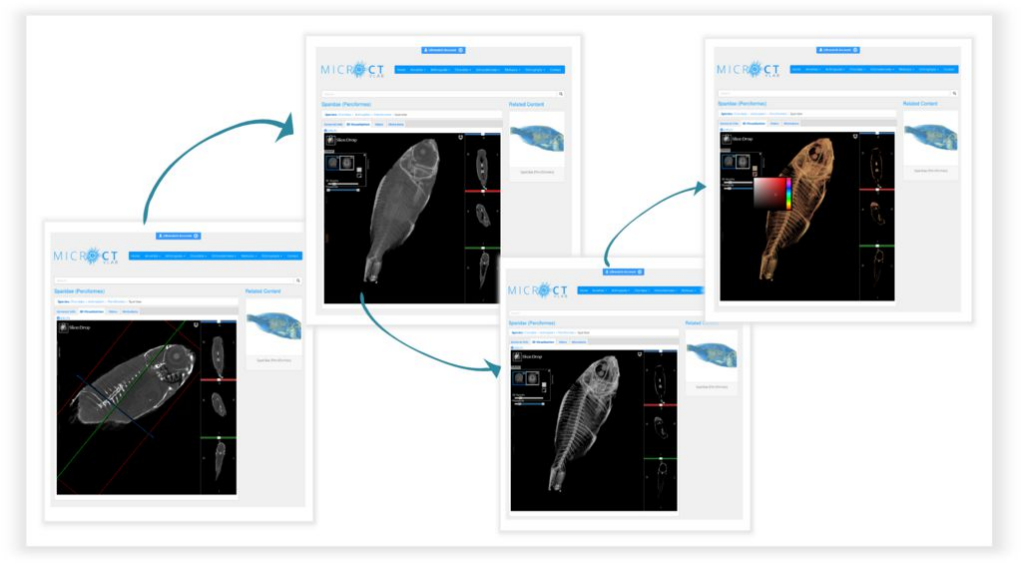
**The Slice:Drop software operation through Micro-CT_vlab_.** Volume rendering of a specimen changing several parameters in the Slice:Drop software.

Using the **3D visualisation** tab (Fig. 2B), the micro-CT dataset can be displayed in 3D using the Slice:Drop software. This software is a web-based 2D/3D viewer using WebGL and HTML5 Canvas to perform volume rendering in a web browser (Haehn 2013). Based on the open source XTK toolkit, Slice:Drop software runs on computers and mobile devices and supports several 3D image file formats. Using the software, the user can either explore the micro-CT dataset in 2D and view all the micro-CT slices in three orthogonal views (x, y, z axes) or, by selecting the 3D icon in the volume tab, the 3D volume of the specimen is displayed. The user has the ability to rotate this 3D specimen and to change the opacity (transparency parameters), the thresholding parameters (to "compress" the grayscale values so that only denser parts or all parts are visible) or to colour the specimen and thus create contrasts with the different colours. The Slice:Drop software provides the on-the-fly rendering of the micro-CT dataset but it does not allow the user to export the screenshot of the specimen. An example of the use of the 3D visualisation tab is presented in Fig. 3.

The **Video** tab displays a short preview video as a demonstration of the specific micro-CT dataset (Fig. 2C), featuring the morphology and anatomy of the specimen.

The **Metadata** tab contains additional information about the dataset, such as contrast enhancement methods, scanning parameters or the creator of the dataset (Fig. 2D). This information is retrieved dynamically from the "Data Services" – the central metadata repository of the LifeWatchGreece Infrastructure. Although very detailed metadata parameters are recorded during the creation of each dataset, not all of these are currently being displayed through the Micro-CT_vlab_, as the data flow to and from the central metadata repository is still under development. Further information about the metadata terms used, the annotation workflow and data management through this semantic model can be found in Faulwetter et al. 2015

**Figure 4.**
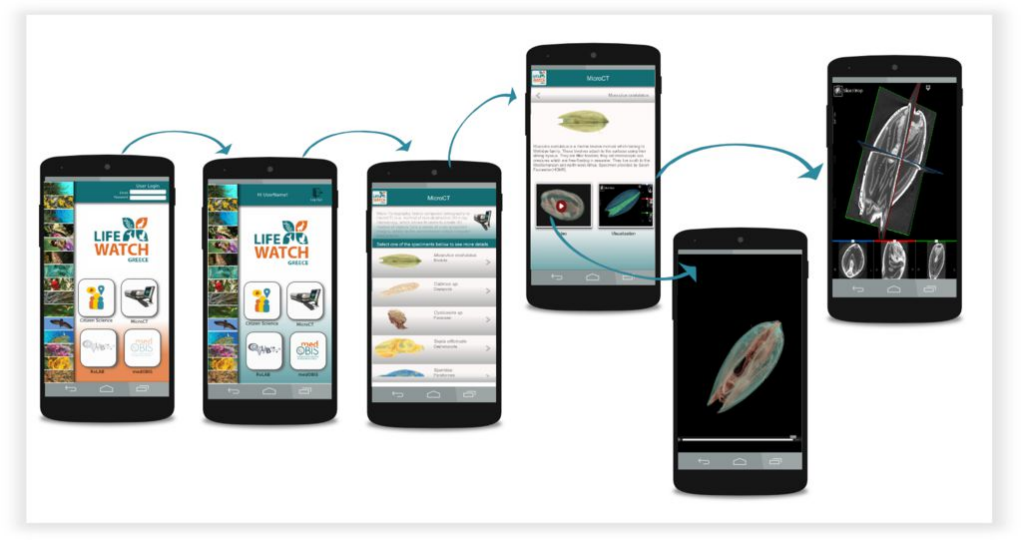
Micro-CT mobile/tablet application and its functionalities.

**Figure 5.**
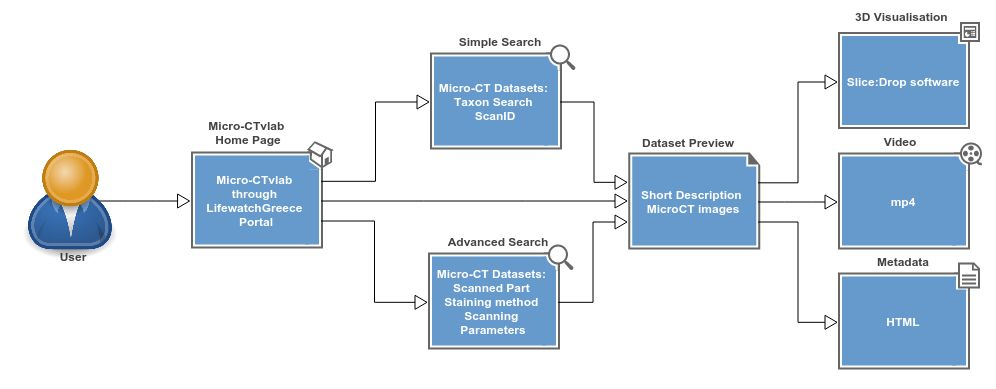
Schematic representation of a user interacting with the Micro-CT_vlab_ web application.

**Figure 6.**
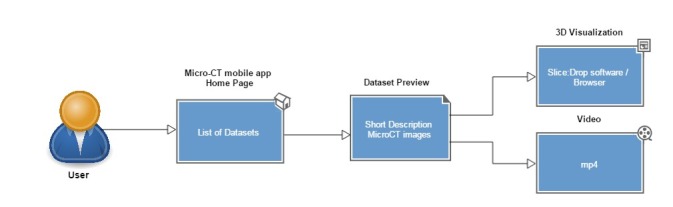
Schematic representation of a user interacting with the Micro-CT_vlab_ mobile/tablet application.

The Micro-CT **mobile/tablet application** has functionalities similar to the Micro-CT_vlab_ web application but presents these in a simplified and compact version (Fig. 4). In the main page of the application, datasets are presented as a preview of images along with the title of the dataset. The application furthermore provides images/videos and a short description of each dataset, and the Slice:Drop software can be used through the browser of the mobile device exactly in the same way as in the web application. The display of metadata, however, is not available in the mobile version. The features available in either version are depicted as a user interaction diagram in Fig. 5 and Fig. 6. Fig. 7 demonstrates the features of the Micro-CT_vlab_in the form of a screencast.

*Minimum system requirements*

The Micro-CT_vlab_ web application has been tested and works on all modern browsers: Google Chrome version 48 > / Mozilla Firefox version 44 / Safari version 9 / Opera version 34 / Microsoft Edge 25 / Internet Explorer 11.

To use the interactive 3D software Slice:Drop, the following are required:

- Operating System: Windows XP SP3 and newer / Mac OSX 10.6 and newer / Linux / Chrome OS
- Computer speed and processor: Use a computer with a minimum of 1GB of RAM and a 2GHz processor
- Internet Speed: Along with compatibility and web standards, Canvas has been carefully crafted to accommodate low bandwidth environments. A minimum of 512kbps is recommended.

The mobile application requires iOS 7 and newer or Android 2.3 and newer.

### Funding

This research has been supported by the LifeWatchGreece infrastucture (ESFRI, MIS 384676), funded by the Greek Government under the General Secretariat of Research and Technology (GSRT), National Strategic Reference Framework (NSRF) and the EU FP7 programme SYNTHESYS3 (FP7-312253).

## Web location (URIs)

Homepage: https://microct.portal.lifewatchgreece.eu/

## Technical specification

Platform: Drupal 7.x, Unity3D

Programming language: php, mysql, html, css, javascript, C#

Operational system: Linux Server

Interface language: html5, css, Javascript

## Usage rights

### Use license

Open Data Commons Attribution License

### IP rights notes

The source code for the mobile application is licensed under MIT license. For the web application the source code is licensed under the GNU General Public License. The content of the MicroCT_vlab_ is available under a Creative Commons Attribution License (CC-BY) unless indicated otherwise (see LifeWatchGreece Data Sharing Agreement).

## Implementation

### Implements specification

In order to deploy a web application for scientists that could handle the complex requirements (implementing 3D volume rendering software, Web Services and online media presentation) of a development like the Micro-CT_vlab_, a custom framework was required that can provide a wide range of features for creating and editing content in a simple and user friendly way. Furthermore, open source technologies that could implement these needs were given priority over commercial solutions.

*Technical architecture*

**Figure 8.**
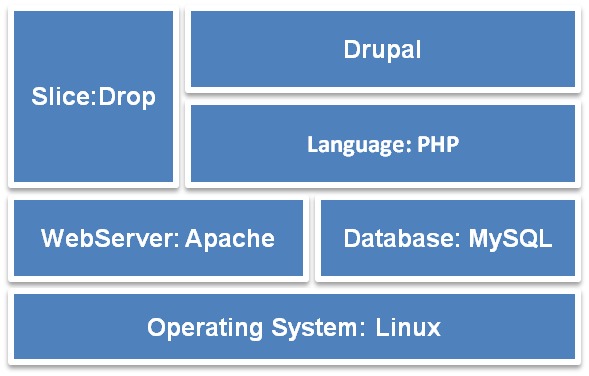
Software Stack: Software that is used by the Micro-CTvlab web application.

The Micro-CT_vlab_ web application has been developed using open source technologies. It is based primarily on the content management system (CMS) Drupal (v.7) and its software stack is illustrated in Fig. 8. Drupal's excellent taxonomy, user, views and content management system with the combination of scalability and flexibility architectures that are provided as out-of-the-box functionalities were the main reasons for choosing this specific open source CMS. Since the LifeWatchGreece portal is likewise based on Drupal, the Micro-CT_vlab_ is seamlessly integrated into the authentication system and user database, as well as all other virtual labs that exist in the portal.

**Figure 9.**
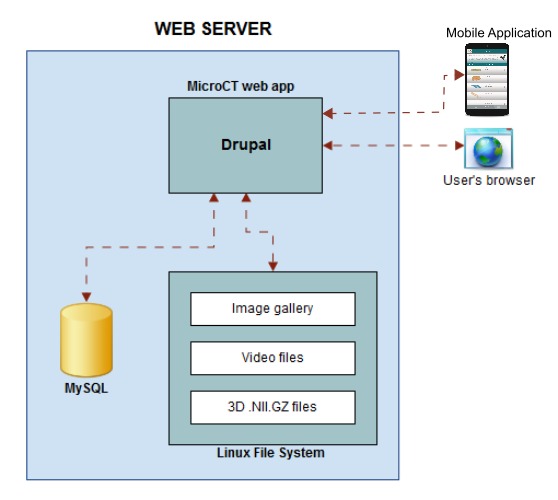
Micro-CT_vlab_: architecture of the web application.

When importing a new dataset, the Micro-CT_vlab_ stores three types of media files (video, images and .nifti files) in the file system and some relevant information in the database (Fig. 9​). A transformation of every micro-CT dataset to .nifti format (3D.NII.GZ) using the Fiji software is needed for Slice:Drop to be able to manage the rendering propertly. Each dataset supports an image gallery with a potentially unlimited number of images in .png and .jpeg image file formats, and one video in .mp4 or .m4v file format.

For the mobile application development, the Unity3D Platform solution has been adopted. The data presented in the mobile application are acquired by https requests to the Drupal portal (Fig. 9). The data are received in json format and after their parsing a list of datasets is extracted. For each dataset the following data fields can be presented: a) one description text, b) one image (the image is downloaded from a url extracted from the json data) c) one video (the url of the video is also extracted from the json data, thus the video is not downloaded but streamed) and d) one url pointing to the Slice:Drop software (the device browser opens the Slice:Drop page of the specific dataset). The mobile application supports Android 2.3 or later version.

### Audience

**Figure 10.**
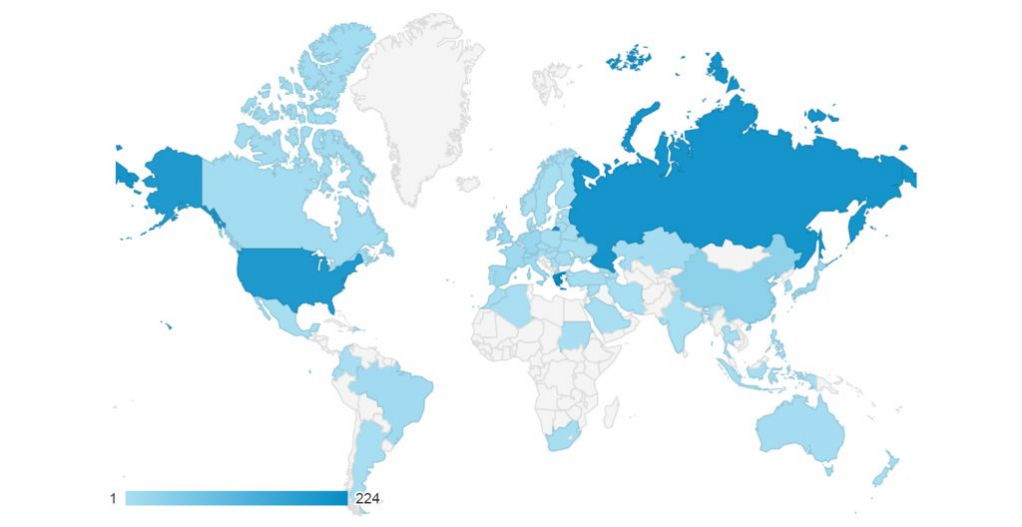
Geographical distribution of unique visitors to the Micro-CT_vlab_ for the period August 2015-February 2016.

The Micro-CT_vlab_ targets a wide audience which includes academics, scientists, students, artists and animators, as well as everyone who is interested in digitisation methods and biological collections or simply wants to explore the 3D visualisations. Already, the tool has attracted a lot of attention after the first months after its launch (Fig. 10).

Experts in micro-CT technology can use the information in the Micro-CT_vlab_ to compare or discover protocols and scanning parameters (best staining solution, usage of filter, scanning voltage, etc.) for different species. Furthermore, members of the scientific community who are not yet familiar with this technology but work in areas such as taxonomy, evolutionary, developmental or functional biology could be attracted by the Micro-CT_vlab_ since this virtual service presents, through a range of examples, the potential for micro-CT imaging in many research fields.

Natural history museums will naturally be highly interested in the Micro-CT_vlab_ and the underlying technology, since there is a need for massive digitisation and dissemination of natural history collections and this virtual lab could be used as a tool to achieve this. Furthermore, the Micro-CT_vlab_ can be used for educational purposes since it offers information on the morphology and anatomy of species and the 3D model scan be interactively manipulated by the students. The simplified mobile/tablet application could attract an even wider audience who are not experts in any of the abovementioned fields but are interested in exploring biological specimens. Several micro-CT scans can be freely downloaded via the Dryad repository and can be used to create 3D surface models which can then be printed in a 3D printer and thus offer an even more tangible experience regarding the anatomy of specimens. All the remaining micro-CT datasets will be available for downloading in the near future (see paragraph "Conclusions and Future Scenario"). Target user groups include museums, aquariums, herbariums, universities and schools, environmental education associations, research institutes, as well as any member of the general public with an interest in natural sciences.

## Additional information

### Conclusions and Future Scenario

The Micro-CT_vlab_ is a web-based virtual lab and a mobile application which presents virtual galleries of 3D micro-CT datasets of biological specimens and innovative online tools for their manipulation and exploration. This is, to our knowledge, an important step towards the massive creation, manipulation and dissemination of three-dimensional morphological datasets. We have developed a standardised workflow for the creation of micro-CT datasets, protocols and terms for documenting each dataset with metadata, and a web-based environment for the publication, dissemination and on-the-fly rendering manipulation of these datasets and their metadata. The Micro-CT_vlab_ in its current stage provides a fully-fledged virtual research enviroment integrating all the above steps. However, developments are ongoing to improve several aspects of the current implementation: a) the large size of the micro-CT datasets constitutes a restriction for native integration and upload of the raw data (i.e. the high-resolution cross-section datasets). Currently, several datasets are available only through external links to the Dryad data repository, but an installation of a storage area network (SAN) is planned to overcome this restriction. With this SAN in place, all datasets will be made available for download; b) a service needs to be developed to allow other micro-CT users to submit and share their raw data through the Micro-CT_vlab_; c) the communication with the LifeWatchGreece data services catalogue needs to be improved to allow refined querying for datasets; d) the process of creating preview files, descriptions and .nifti files for online manipulation needs to be automated so that the integration of additional datasets can be achieved more quickly.

In its current version the Micro-CTvlab is only an initial step for the implementation of the massive digitisation of micro-CT datasets, but it forms the basis for future developments of centralised repositories for such data. The digitisation of natural history collections is rapidly advancing (Blagoderov and Smith 2012), and natural history museums have a responsibility to not only create such cyberspecimens but also to take responsibility in curating and disseminating them. To make specimens continuously and simultaneously available to the research community, virtual collections such as those in the Micro-CTvlab will need to be deployed more massively, and these virtual collections will need to comprise mechanisms for an extensive documentation of the cyberspecimens so that they are searchable and retrievable (Faulwetter et al. 2013, Stoev et al. 2013). Furthermore, features such as preview images/ videos, descriptions and tools for the direct interaction with the data are indispensable for the success of such virtual collections for several reasons: a) researchers can understand at a glance the content of the dataset without having to download the high resolution dataset; b) the dataset can be explored online, removing the need for technical knowledge, additional software or a high-speed internet connection and c) a pleasing and easy-to-use presentation of the data will not only address experts but the general public at large.

In the long term these develoments will transform taxonomic research into a true cyber-discipline: more and more morphological and anatomical data will become available in an electronic, shareable format. This will speed up systematic research, as morphological, high-resolution information can be accessed at the click of a mouse, and analysed in completely new ways by pattern recognition algorithms, steering comparative morphology into a new direction and shifting from the current use of phenetics to that of phenomics (Houle et al. 2010).

## Figures and Tables

**Figure 7.** Screencast demonstrating the features of the Micro-CT_vlab._
